# The integration between nonsymbolic and symbolic numbers: Evidence from an EEG study

**DOI:** 10.1002/brb3.938

**Published:** 2018-03-08

**Authors:** Ruizhe Liu, Christian D. Schunn, Julie A. Fiez, Melissa E. Libertus

**Affiliations:** ^1^ Department of Psychology University of Pittsburgh Pittsburgh PA USA; ^2^ Learning Research and Development Center University of Pittsburgh Pittsburgh PA USA; ^3^ Department of Neuroscience University of Pittsburgh Pittsburgh PA USA

**Keywords:** event‐related potentials, integration, nonsymbolic numbers, symbolic numbers

## Abstract

**Introduction:**

Adults can represent numerical information in nonsymbolic and symbolic formats and flexibly switch between the two. While some studies suggest a strong link between the two number representation systems (e.g., Piazza, Izard, Pinel, Le Bihan, & Dehaene, 2004 *Neuron*, 44(3), 547), other studies show evidence against the strong‐link hypothesis (e.g., Lyons, Ansari, & Beilock, 2012 *Journal of Experimental Psychology: General*, 141(4), 635). This inconsistency could arise from the relation between task demands and the closeness of the link between the two number systems.

**Methods:**

We used a passive viewing task and event‐related potentials (ERP) to examine the temporal dynamics of the implicit integration between the nonsymbolic and symbolic systems. We focused on two ERP components over posterior scalp sites that were found to be sensitive to numerical distances and ratio differences in both numerical formats: a negative component that peaks around 170 ms poststimulus (N1) and a positive component that peaks around 200 ms poststimulus (P2p). We examined adults' (*n *=* *55) ERPs when they were passively viewing simultaneously presented dot quantities and Arabic numerals (i.e., nonsymbolic and symbolic numerical information) in the double‐digit range. For each stimulus, the nonsymbolic and symbolic content either matched or mismatched in number. We also asked each participant to estimate dot quantities in a separate behavioral task and observed that they tended to underestimate the actual dot quantities, suggesting a need to adjust the match between nonsymbolic and symbolic information to reflect the perceived quantity of the nonsymbolic information.

**Results:**

Using this adjustment, participants showed greater N1 and P2p amplitudes when perceived dot quantities matched Arabic numerals than when there was a mismatch. However, no differences were found between the unadjusted match and mismatch conditions.

**Conclusion:**

Our findings suggest that adults rapidly integrate nonsymbolic and symbolic formats of double‐digit numbers, but evidence of such integration is best observed when the perceived (rather than veridical) dot quantity is considered.

## 1 INTRODUCTION

Even though we use mathematics frequently in our daily lives, it is unclear how the knowledge that is required to perform math is represented in the brain. Previous research has shown that people have access to two systems representing numerical information: One is an approximate number system (ANS) that represents the numerical magnitudes from nonsymbolic numbers >4 (e.g., a dot quantity containing 23 dots as “twenty‐ish”); the other is a symbolic number system (SNS) that allows for the representation of exact numerical information provided by symbolic numbers (e.g., Arabic numerals or number words). While these two systems differ fundamentally in their representational capacities, there is evidence to suggest that they are also integrated. This study aimed to investigate the context in which such integration occurs at the neural level and its underlying temporal dynamics.

### 1.1 Features of the approximate and symbolic number systems

The acuity of the ANS is typically measured using a nonsymbolic number comparison task in which people are presented with two dot quantities in different colors and asked which color has more dots (Dietrich, Huber, & Nuerk, 2015). The dots are presented too briefly for people to count. Therefore, people have to estimate the dot quantities to make a judgment. The visual perceptual cues such as surface area and dot size are commonly controlled so that the number of dots is the only consistent cue across trials. By changing the ratio between the smaller and larger dot quantities in the comparison task, it can be shown that response times and accuracies vary as a function of ratio. For example, if the magnitude of the ratio is large, for example, four dots versus eight dots (1:2 ratio), responses tend to be fast and precise, which indicates that a large ratio makes the comparison easy. If the magnitude of the ratio is small, for example, 15 dots versus 16 dots (15:16 ratio), responses tend to be slower than in the easy ratio condition, and the accuracy is typically lower (Barth, et al., 2003; Cordes, Gelman, Gallistel, & Whalen, 2001; Pica, Lemer, Izard, & Dehaene, 2004), indicating harder comparison. Converging evidence from developmental and comparative studies as well as studies with people whose languages do not have number words shows ratio‐dependent performance on nonsymbolic number comparison tasks suggesting a key feature of the ANS: independence from language (Cantlon, Brannon, Carter, & Pelphrey, 2006; Izard, Sann, Spelke, & Streri, 2009; Libertus and Brannon, 2009; Lipton & Spelke, 2003; Nieder, 2009; Pica et al., 2004; Xu & Spelke, 2000).

Unlike the ANS, the development of the SNS has a later onset and continues to develop into adulthood. The acquisition of symbolic numbers starts with learning to recite number words around 2 years of age, with gradually increasing understanding of their meaning (Fuson, 2012; Wynn, 1990). Building upon the basic symbolic knowledge, children learn conceptual and procedural knowledge of basic arithmetic and other advanced math knowledge, such as algebra and calculus through both informal and formal math instruction later in life. In stark contrast to the ANS, the SNS thus requires language and an understanding of a formal symbol system.

### 1.2 The mapping between the approximate number system and the symbolic number system

Mixed and indirect evidence for a link between the ANS and the SNS comes from number comparison tasks involving symbolic number stimuli. On one hand, it has long been established that when comparing two symbolic numbers, people's responses are slower when the numerical difference, also known as the numerical distance, between two numbers decreases (Dehaene, Dupoux, & Mehler, 1990; Moyer & Landauer, 1967). For instance, judging 5 is smaller than 9 is easier than judging 5 is smaller than 6. This effect is known as the distance effect, and its existence suggests that symbolic number comparisons activate corresponding nonsymbolic number representations because purely symbolic representations of 5 and 9 should be as discriminable as 5 and 6. On the other hand, other variants of symbolic number comparison tasks have dampened the idea of an integration between the ANS and the SNS. Lyons et al. (2012) asked adults to perform numerical comparison tasks in which two numbers could be both nonsymbolic, both symbolic, or one nonsymbolic and one symbolic. They found that the performance in the mixed‐formats condition was worse than the performance in the other two single‐format conditions no matter whether the two numbers were presented simultaneously or sequentially. They attributed the decrement in performance in the mixed‐formats condition to a weaker integration between nonsymbolic and symbolic numbers compared to the within‐format integration.

Another way of assessing the link between the ANS and the SNS is via nonsymbolic number estimation tasks. In a typical nonsymbolic number estimation task, people are presented with a bunch of dots and are asked to estimate how many dots there are. The dot quantities are presented too briefly for them to count, and people have to rely on their nonsymbolic number representations to make their judgments. Meanwhile, people also need to retrieve information from their symbolic number knowledge in order to give their verbal estimation. Typically, people have precise estimates for small numbers, such as 4 and 5. As the numbers get bigger, there is increasingly more variation in people's estimates (Dehaene, Izard, Spelke, & Pica, 2008; Revkin, Piazza, Izard, Cohen, & Dehaene, 2008). For example, when there are six dots in a display, people's answers are more likely to be five, six, or seven dots. It is less likely for them to say that there are 20 dots. However, when there are 60 dots in a display, people's answers tend to vary even more, for example, from 40 to 80.

More critically, previous research found that people tend to underestimate large quantities in nonsymbolic number estimation tasks (Crollen, Castronovo, & Seron, 2011; Izard & Dehaene, 2008; Krueger, 1982; Odic, Im, Eisinger, Ly, & Halberda, 2015). For example, when presented with 60 dots, people more commonly estimate fewer than 60 dots in contrast to estimating more than 60 dots. In one early study, a large sample of adults was presented with only a single trial (i.e., one dot quantity) ranging from 25 to 300 dots and was asked to estimate the number of dots (Krueger, 1982). Underestimation bias was observed for all dot quantities >30. This underestimation bias has three important aspects. First, the degree of the underestimation increases as the quantity increases (e.g., Poulton, 1968, 1975, 1979). In other words, the difference between a dot quantity and its estimate is greater if the quantity is large compared to when it is small. Second, the underestimation bias can be calibrated by being exposed to a reference quantity. In one study, adult participants were shown a dot quantity labeled with “30” before a dot estimation task. The reference dot quantity either contained 25, 30, or 39 dots, which correspondingly induced overestimation, linear‐like estimation, and underestimation in participants' performance (Izard & Dehaene, 2008). Third, there are individual differences in the underestimation bias in adults (Izard & Dehaene, 2008; Odic et al., 2015) and young children who have acquired symbolic number knowledge (Libertus, Odic, Feigenson, & Halberda, 2016). Altogether, these behavioral findings suggest that people are able to map between the ANS and SNS, but that this mapping is not precise and is subject to a systematic underestimation bias. Importantly, the behavioral evidence is unclear whether this mapping is automatic or only exists when people are forced to provide an exact label for a nonsymbolic quantity.

In addition to these behavioral studies, brain imaging studies provide evidence of the mapping between the ANS and the SNS, suggesting that the parietal lobe is important for both. Using event‐related potentials (ERPs), the P2p component, a positive component over posterior parietal scalp sites which peaks around 200 ms after stimulus onset, was found to be sensitive to the distance effect in both nonsymbolic and symbolic number comparison tasks (Dehaene, 1996; Libertus, Woldorff, & Brannon, 2007; Temple & Posner, 1998). Specifically, the amplitude of the P2p was greater for small distances than large distances. Other studies using different paradigms confirmed this finding (Hsu & Szücs, 2012; Hyde & Spelke, 2009; Rubinsten, Dana, Lavro, & Berger, 2013). In fMRI studies, the IPS was repeatedly found to be activated in nonsymbolic and symbolic number comparison tasks (Ansari, Garcia, Lucas, Hamon, & Dhital, 2005; Fias, Lammertyn, Reynvoet, Dupont, & Orban, 2003).

However, one critical aspect of the behavioral, ERP and fMRI studies reviewed above is that they all required participants to make explicit judgments about numbers and/or dot quantities. To examine whether a link between the ANS and the SNS depends on explicit numerical judgments, it is necessary to use non‐numerical tasks or no task at all. A recent behavioral study (Liu, Schunn, Fiez, & Libertus, 2015) took a step in this direction using a number decision task that was similar to a lexical decision task for word‐like stimuli. In this number decision task, participants were briefly shown an image that contained either an Arabic numeral (two‐digit number) or a letter pair and they were instructed to judge whether they saw a valid numeral (i.e., two digits) or not. The numeral/letter pairs were superimposed on top of a dot quantity, which the participants could ignore for the number decision task. The number of dots either matched or mismatched with the Arabic numeral. Participants' accuracy and response times were better for the match trials than the mismatch trials in the Arabic numeral condition suggesting that even without explicit judgments about numerical magnitudes, participants associated the nonsymbolic and symbolic numerical information.

In another study that did not require explicit numerical judgments, brain activation was measured via fMRI as adults were adapted to numbers in either nonsymbolic or symbolic format and tested with same‐format or cross‐format novel numbers (Piazza et al., 2004). It was found that in the right IPS, the blood oxygen level‐dependent (BOLD) signal recovery after the presentation of the novel numbers was dependent on numerical distance between the adapted number and the novel number but invariant to number formats. The findings imply that the human brain can automatically pick up numerical information in different formats and integrate it. However, the BOLD signal recovery in the left IPS was dependent on both numerical distance and number formats suggesting that the left hemisphere does not automatically integrate information across the ANS and the SNS.

Studies that compared the more detailed brain activation patterns for nonsymbolic numbers and symbolic numbers found that there was not much overlap between the two formats. For example, one fMRI study (Eger et al., 2009) examined participants when they were presented with either nonsymbolic or symbolic numbers. A multivoxel pattern analysis that used classifiers to identify different activation patterns of different quantities within one format in IPS revealed high classification accuracies (~77%) in the nonsymbolic format compared to the symbolic format (accuracies were ~57%). The classification generalization was poor from one format to another. A classifier trained to differentiate quantities within one format (e.g., Arabic numeral) could not differentiate as well between quantities presented in another format (e.g., nonsymbolic numbers). Similar results of classification accuracies as well as generalization were found in other fMRI studies (Bulthé, De Smedt, & de Beeck, 2014, 2015; Lyons, Ansari, & Beilock, 2015), suggesting that even though IPS is responsive to numerical information in general, nonsymbolic and symbolic numbers are not represented in the same way in IPS.

As reviewed above, previous findings provide mixed evidence regarding the integration between the ANS and the SNS. In addition, all of these studies used fMRI, which limits the conclusions that can be drawn from these results. First and foremost, fMRI does not provide a good temporal resolution of the underlying brain activity. Thus, it is possible that more subtle, short‐lived neural signals of integration remain unnoticed. Second, the range of numerical stimuli was limited, which might have artificially created an illusion of integration. In the fMRI studies that used classification methods to examine nonsymbolic and symbolic number representations in the IPS (Bulthé et al., 2014, 2015; Eger et al., 2009; Lyons et al., 2015), the number ranges were small and mostly under 10. Piazza et al. (2004) used larger numbers, but the number stimuli were rather categorical (small vs. large) instead of continuous. Besides, the participants in this study were familiarized with example dot quantities of each category and were told the true approximate ranges before the scan sessions, potentially affecting numerical integration.

Here, we designed a passive viewing EEG task, in which the two formats of numbers (dot quantities and Arabic numerals) were simultaneously presented to participants without an explicit number‐related task. In addition, we included a large, continuous range of numbers. Similar to the stimuli used by Liu et al. (2015), the nonsymbolic number either matched or mismatched with the symbolic numbers. In line with previous studies, we examined two ERP components over posterior scalp sites that are thought to reflect number processing: the N1, the first negative component peaking around 150 ms poststimulus, and the P2p, the second posterior positivity peaking around 200 ms poststimulus (Dehaene, 1996; Hyde & Spelke, 2009; Libertus et al., 2007; Rubinsten et al., 2013; Temple & Posner, 1998). Furthermore, as the mental representation of nonsymbolic numbers is expected to be imprecise and subject to systematic estimation biases, we administered a nonsymbolic number estimation task. We hypothesized that the N1 and P2p amplitudes would show stronger differences between numerical matches and mismatches after adjusting for participants' estimation bias than without the adjustment, as previously found in a behavioral study (Liu et al., 2015).

## 2 METHODS

### 2.1 Participants

Sixty‐four participants (mean age = 19.3 ± 1.5 years, 34 females, 61% White, 30% Asian, 3% African American, 6% Other) were recruited from the University of Pittsburgh subject pool and received course credits for their participation. All participants provided written informed consent before participating in accordance with the Declaration of Helsinki and a protocol approved by the local Institutional Review Board. Data from nine participants were excluded because of low quality of behavioral data (i.e., random responding in the behavioral task, *n = *3), excessive EEG artifacts (i.e., more than 50% trials in the EEG task being rejected as artifacts, *n *=* *5), or failing to complete the EEG (font‐change detection) task (*n *=* *1). After exclusions, 55 participants remained in the behavioral and ERP analysis.

### 2.2 Stimuli and tasks

#### 2.2.1 Behavioral nonsymbolic number estimation task

The estimation task was identical to the nonsymbolic number estimation task used by Liu et al. (2015). Briefly, each stimulus consisted of a 400‐by‐400 pixel image comprising a black dot quantity with a superimposed, translucent blue, double‐digit Arabic numeral, or two random capital letters. The font of all numerals and letters was set as Arial Black. The background color of the images was white, and the background color of the screen was black. We selected 12 Arabic numerals with a range from 11 to 63, 12 letter pairs, and 24 dot quantities. The dot quantities and their respective pairings with Arabic numerals or letter pairs are listed in Table 1. A script created by Dehaene, Izard, and Piazza (2005) generated the dot arrays, with half of the images equated on the individual dot size and the other half of the images equated for the cumulative surface area of all dots to avoid consistent correlations between perceptual features and dot quantities. We generated six variations of each quantity with respect to the layout and size (three different sizes and two layouts). Dots were randomly localized within the 400‐by‐400 pixel area to generate different layouts. However, density was not controlled when generating different layouts. There were 144 dot arrays in total. For each Arabic numeral and corresponding letter pair, three categories of images were created: match with dot quantity, mismatch with dot quantity where dot quantity < Arabic number, and mismatch with dot quantity where dot quantity > Arabic number. In the case of mismatches, the ratio between the dot quantity and Arabic numeral was always 1.5 (i.e., 3:2 or 2:3). Considering the six variations of each dot quantity, for each Arabic numeral or letter pair there were 18 images. In total, 432 images were created, half of them as dots with Arabic numerals and the other half as dots with letters.

**Table 1:** Arabic numerals, letters, and dot quantities used in each match and mismatch condition in the behavioral nonsymbolic estimation task and the symbolic integration EEG task

Behavioral nonsymbolic estimation task				Symbolic integration EEG task					
Arabic numeral (Match)	Letter	Mismatch Dot < Num	Mismatch Dot > Num	Arabic Numeral (Match)	Perceived dot quantity	Mismatch Dot < Num	Perceived dot quantity	Mismatch Dot > Num	Perceived dot quantity
11	RC	7	17	6	8.61	4	6.52	9	11.43
13	PH	9	20	7	9.59	5	7.60	10	12.31
17	CF	11	26	8	10.52	5	7.60	12	13.99
21	LR	14	32	9	11.43	6	8.61	14	15.61
25	QX	17	38	28	25.65	18	18.67	42	34.45
28	GM	19	42	29	26.31	19	19.41	44	35.64
32	KJ	21	48	31	27.61	20	20.13	47	37.41
38	XR	25	57	32	28.26	21	20.85	48	37.99
42	YG	28	63	34	29.53	23	22.26	50	39.14
48	JD	32	72	36	30.78	24	22.95	53	40.86
59	PN	39	89	38	32.02	24	22.95	60	44.77
63	FW	42	95	39	32.63	25	23.64	61	45.31
				41	33.85	27	24.99	62	45.86
				42	34.45	28	25.65	63	46.41
				44	35.64	30	26.97	64	46.95
				46	36.82	31	27.61	69	49.64

Right panel: The first column represents the Arabic numeral as well as the dot quantities used to create the match trials. The 3rd and 5th column represent the dot quantities used to create the mismatch trials. The perceived dot quantity of each actual dot quantity used in the symbolic integration EEG task was calculated (1) using each participant's best fitting power function to calculate the perceived dot quantity and (2) average the perceived dot quantity for each actual dot quantity across all participants.

Participants were instructed to estimate the quantity of dots shown in the image, ignoring the numerals and letters, type in their answer, and hit the Enter key to move on to the next trial. Each image was presented for 400 ms, followed by a blank response screen until the participants responded. Although there was no time limit for participants to type in their answer, they were encouraged to respond as quickly and accurately as possible. The entire task contained six blocks with 72 trials, each separated by five short breaks. The entire task took about 40 min to complete.

#### 2.2.2 Symbolic integration task with EEG acquisition

The stimuli were identical to those used in the behavioral nonsymbolic number estimation task, except that there were no letter trials and we extended the Arabic numerals and corresponding dot quantities to cover single digit numerals and more numerals in the 30–40 range. The latter change was designed to create a more balanced “match” and “mismatch” set after accounting for a range of possible estimation biases. A complete list of all Arabic numerals and the corresponding mismatch quantities can be found in Table 1. As in the behavioral non‐symbolic number estimation task, the dot quantity either matched or mismatched the Arabic numeral. Also, in the mismatch condition, half of the images were dot quantities less than the Arabic numerals and half of the images were dot quantities greater than the Arabic numerals. Aside from these images, 27 images with the same Arabic numerals but different font (Marker Felt) were created for an orthogonal font‐change detection task to encourage participants' attentiveness to the stimuli, but avoid explicit number processing. However, data from the trials with these images were not included in any analysis. The total set of 315 images was repeated four times and thus created a set of 1,260 trials for the entire task.

Each trial started with a fixation cross centered on the screen for an average duration of 500 ms (range: 450–550 ms). Immediately after the fixation disappeared, a stimulus image was presented for a fixed 500 ms duration, followed by a fixed 250 ms fixation. Participants were instructed to look at the images and press a key on a keypad when they detected a font change in the Arabic numeral (11% of all trials). The entire task contained six blocks with 210 trials each separated by five short breaks. The entire task took about 30 min to complete.

EEG data were recorded throughout the symbolic integration task using a 64‐channel Brain Vision actiChamp system (Brainproducts, Munich, Germany). The sampling rate was set at 1,000 Hz during recording and was resampled at 500 Hz offline. The impedance of all electrodes was kept under 5 kΩ. Electrodes were referenced to the right mastoid during recording and later algebraically re‐referenced to an average of the right and left mastoids during offline analysis.

### 2.3 Procedure

After obtaining written consent, while the experimenters set up the EEG equipment and adjusted the impedance of the electrodes, the participants were given the behavioral nonsymbolic number estimation task. After they finished the estimation task, they were given a short break, followed by the symbolic integration task with EEG acquisition. Finally, they were given a short demographic questionnaire asking about age, gender, major, year in college, and the number of math classes taken in high school and college.

### 2.4 Data analysis

#### 2.4.1 Behavioral nonsymbolic number estimation task

To determine whether participants were using estimation instead of counting, we performed a repeated‐measures anova and correlation analyses to examine the relation between the dot quantities that the participants saw and their responses. If participants were paying attention to the task and were responding reasonably, their estimate would increase as the dot quantities increase. Moreover, as the dot quantities increase, the variability of participants' estimates should also increase (Odic et al., 2015).

As the nonsymbolic number estimation task included both number and letter trials, we first compared participants' estimates on these trials types. Each participant's estimates from the number and letter trials in the behavioral nonsymbolic number estimation task were fitted using separate power functions in PsiMLE 1.0 (Odic et al., 2015). PsiMLE is an R‐based package that uses a maximum‐likelihood estimation approach to optimize the parameters of power functions that capture participants' behavioral responses in the nonsymbolic number estimation task. Specifically, PsiMLE estimates the scaling factor α, the exponent β of a power function *y* = α*x* ^β^, and an extra parameter σ that describes the variability of the estimates of each dot quantity given the actual dot quantities *x* and each participant's responses *y* with the likelihood function $L\left(\alpha ,\beta ,\sigma |x,y\right)=\prod_{i=1}^{n}{\sqrt{\left(2\pi \alpha *x^{\beta }*\sigma \right)}}^{-1}\exp\left(-\frac{1}{2}{\left(y-\alpha *x^{\beta }\right)}^{2}{\left(\alpha *x^{\beta }*\sigma \right)}^{-2}\right).$Repeated‐measures anovas were used to examine whether the exponents and scaling factors from number trials differed from the exponents and scaling factors from letter trials. There were no significant differences between the exponents and scaling factors in the number and letter trials (exponent β: *F*(1,54) = 1.29, *p *=* *.26; scaling factor α: *F*(1,54) = 2.44, *p *=* *.12). To examine the reliability of the behavioral estimation task, we also submitted the exponents and scaling factors to a correlational analysis. There were very high positive correlations for both exponents and scaling factors across the number and letter trials (exponent β: *r*(1,60) = .88, *p *<* *.001; scaling factor α: *r*(1,60) = .93, *p *<* *.001). Hence, we collapsed across number and letter trials and fitted power functions for all trials for each participant. These power functions were used to adjust for the perceived dot quantities used in the symbolic integration EEG task (see below).

#### 2.4.2 Symbolic integration EEG task

Raw EEG data were processed offline in EEGLab (Delorme & Makeig, 2004) and ERPLab (Lopez‐Calderon & Luck, 2014). The data were filtered at 0.1–60 Hz. Four artifact rejection algorithms were used to reject trials with eye blinks, horizontal eye movements, motion, electromyography (EMG), and other noises: simple voltage threshold detection, peak‐to‐peak threshold detection, blink detection, and step‐like artifact detection. The rejection threshold for each algorithm was manually set and adjusted slightly based on each participant's data because their overall signal strengths varied substantially. The range of the thresholds was 80 to 120 μV. For participants whose general signal voltage was low (e.g., 90 μV), we used a threshold close to the lower boundary (close to 80 μV) and vice versa. After artifact rejection, the EEG data were segmented into 700‐ms segments consisting of a 200‐ms baseline prior to stimulus onset and 500 ms after stimulus onset. Segmented EEG data were selectively averaged with respect to each pair of dot quantity and Arabic numeral to create ERPs. ERPs were first grouped into match and mismatch conditions according to actual dot quantities, as described in Table 1. Note that the trials in the embedded font‐change detection task were excluded from EEG data analysis but were used to determine whether participants were attending to the stimuli during EEG recording.

Because participants showed large underestimation biases on the behavioral nonsymbolic number estimation task, we also adjusted the match and mismatch conditions based on each participant's best fitting power function derived from the behavioral estimation task. For instance, an image that contained 42 dots and the Arabic numeral “28” was originally considered a “mismatch” while an image that contained 42 dots and the Arabic numeral “42” was considered a “match” before the adjustment. For a participant whose power fitting function revealed an estimate of 42 dots as 28 dots, the 42 dots‐28 numeral image was labeled as “match” whereas the 42 dots‐42 numeral image was labeled as “mismatch” after the adjustment. To control for perceptual features of the dot quantities, we only used adjusted mismatch trials that contained the same dot quantities as the adjusted match trials in the adjusted ERP analysis. Based on previous work (Hyde & Spelke, 2009; Libertus et al., 2007), we selected two ERP components of interest: N1 (130–200 ms), and P2p ERP components (200–250 ms). As ERPs are not highly informative of spatial information related to brain activation, we focused on the overall brain response pattern over a relative large brain region that covers several electrodes. Specifically, we selected four electrodes of interest over posterior parietal scalp sites from each hemisphere to obtain enough coverage of the posterior regions that have been reported in previous studies (Hyde & Spelke, 2009; Libertus et al., 2007). Electrodes P3, P5, PO3, and PO7 were selected from the left hemisphere, and electrodes P4, P6, PO4, and PO8 were selected from the right. By averaging across each set of four electrodes, two regions of interest for each hemisphere (ROI_L_ and ROI_R_) were formed. The mean amplitude of each component at each of the two ROIs was exported from ERPLab for both the no‐adjustment analysis and the after‐adjustment analysis. A two‐way (Trial Type, Hemisphere) repeated‐measures anova was run for overall main effects and interactions separately for the N1 and P2p components and separately for unadjusted and adjusted data.

## 3 RESULTS

### 3.1 Behavioral results

#### 3.1.1 Nonsymbolic number estimation task

We first examined participants' performance in the estimation task to obtain personalized quantity estimation parameters to use in the ERP analysis. For each participant, we removed estimates that were more than three standard deviations from their average estimate across all trials to remove extreme estimates (e.g., 500) from the data that most likely resulted from typing errors. To show that the participants followed the task instruction and estimated dot quantities instead of randomly inputting responses, we conducted two correlation analyses on the quality of the estimates. We found a very high correlation in all participants, mean correlation coefficient *r *=* *.97, standard deviation (*SD*) = 0.02, between the actual dot quantities that were presented and participants' estimates. We also found a positive correlation, mean correlation coefficient *r *=* *.68, *SD *= 0.20, between the actual dot quantities and the variability in participants' estimates, which is consistent with participants relying on their approximate number system when making their estimates.

**Figure 1:**
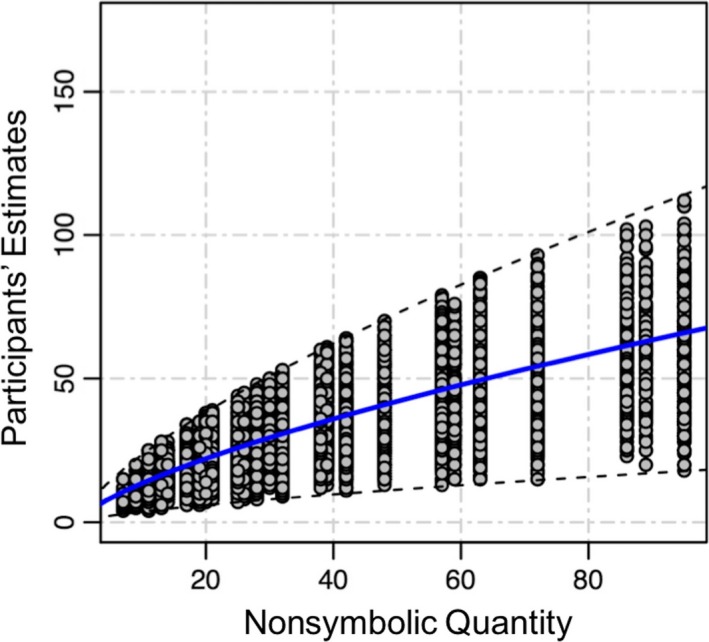
The relation between nonsymbolic numerosities presented in the behavioral estimation task and participants' mean estimates. The blue line represents the mean power fitting function, *Y *= 2.72 * *X* ^0.70^, where *Y* is the predicted perceived dot quantity and *X* is the actual presented dot quantity. The dashed lines represent upper boundary and lower boundary of outlier removal

Next, we fitted each participant's estimates with the best fitting power function and obtained the corresponding exponents and scaling factors. We first removed outliers from the estimates of each dot quantity for each participant. For all the trials with the same dot quantity, the estimates that were more than three standard deviations above or below the mean were removed. The mean exponent was 0.70, *SD *= 1.3. The mean scaling factor was 2.72, *SD *= 0.14. Each participant's estimates of the dot quantities as well as the mean power fitting curve are shown in Figure 1. As shown in this figure, participants exhibited a strong underestimation bias, especially for larger dot quantities. We used each participant's best fitting power function to calculate the person's perceived dot quantity of each actual dot quantity. Then, we used these estimates to calculate the average perceived dot quantities across our entire sample that are listed in Table 1 (right panel, columns 2, 4, and 6).

#### 3.1.2 Symbolic integration EEG task

We analyzed participants' performance in the font‐change detection task that was embedded in the symbolic integration EEG task. The mean response time was 449 ms, *SD *= 16 ms. The mean accuracy was 95%, *SD *= 6%. Hence, the participants responded to the stimuli with a different font quickly and accurately, which confirms that they were engaged in this (non‐numerical) task through the entire EEG recording.

### 3.2 ERP results

**Figure 2:**
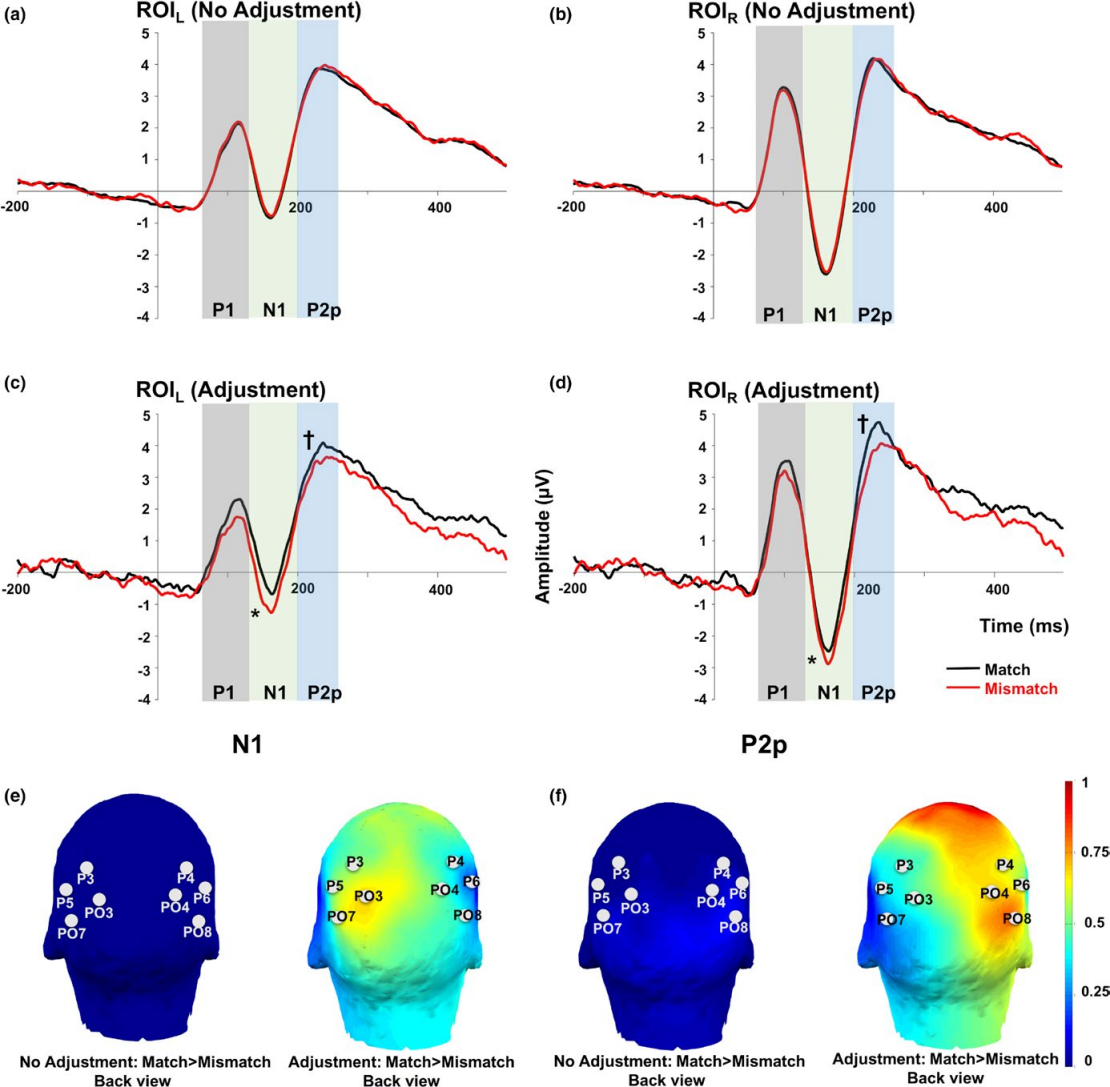
The ERP waveforms of match (black lines) and mismatch condition (red lines). (a) The waveforms of the left ROI in the no‐adjustment analysis. (b) The waveforms of the right ROI in the no‐adjustment analysis. (c) The waveforms of the left ROI in the adjustment analysis. (d) The waveforms of the right ROI in the adjustment analysis. Gray bars: P1 (70–130 ms). Light green bars: N1 (130–200 ms). Light blue bars: P2p (200–250 ms). (e, f) Topographic map of the mismatch effect for N1 component (e) and P2p component (f). Left: unadjusted data. Right: adjusted data. *p < .05. †*p *< .1

Our analyses focused on two ERP components that have been previously established in the literature (Libertus et al., 2007; Temple & Posner, 1998): N1 (130–200 ms) and P2p (200–250 ms). In line with previous studies, we concentrated on two ROIs over bilateral occipito‐parietal scalp sites (Hyde & Spelke, 2009; Libertus et al., 2007). Unadjusted and adjusted match and mismatch ERP waveforms for both ROIs are shown in Figure 2. For unadjusted and adjusted data in the N1 and P2p time window, we conducted a repeated‐measures anova with Trial Type (match vs. mismatch) and Hemisphere (left vs. right) as repeated factors (see Table 2 for the mean and standard deviation of the ERP amplitudes of each ROI of each Trial Type before and after the adjustment).

**Table 2:** The Trial Type effect on the N1 and P2p component in the adjusted and unadjusted analyses

ERP component	Adjustment	Condition	Mean	*SE*	*F*	*n*	*p*	η^*2*^
N1 (130–200 ms)	Unadjusted	Match	3.71	0.30	1.67	55	.20	0.03
		Mismatch	3.22	0.33				
	Adjusted	Match	−0.19	0.37	4.93	55	.03*	0.084
		Mismatch	−0.647	0.33				
P2p (200–250 ms)	Unadjusted	Match	3.41	0.27	1.275	55	.26	0.023
		Mismatch	3.52	0.28				
	Adjusted	Match	3.71	0.30	3.03	55	.087†	0.053
		Mismatch	3.22	0.33				

^*^ *p *< .05; ^†^ *p *< .1.

### 3.3 Without adjustment

#### 3.3.1 N1 (130–200 ms)

The repeated‐measures anova showed a significant main effect of Hemisphere (*F*(1,54) = 32.25, *p *<* *.001, η^2 ^= 0.37). When averaging across the two trial types, the ROI_L_ had higher ERP amplitudes than the ROI_R_ (left: *M *=* *0.28 μV, standard error of mean *(SE) *= 0.33; right: *M *=* *−1.03 μV, *SE *= 0.37). No other main effects or interactions were significant (*ps *> .2).

#### 3.3.2 P2p (200–250 ms)

There were no significant main effects or interactions in the Trial Type by Hemisphere repeated‐measures anova (all *ps *> .26).

### 3.4 With adjustment

#### 3.4.1 N1 (130–200 ms)

There was a significant difference between the ERP amplitudes in the ROI_L_ and ROI_R,_ *F*(1,54) = 25.81, *p *<* *.001, η^2 ^= 0.323. The ERP amplitudes in the ROI_L_ were higher than the ERP amplitudes in the ROI_R_ (left: *M *=* *1.75 μV, *SE *= 0.28; right: *M *=* *1.29 μV, *SE *= 0.27). We also found a significant difference between adjusted match and mismatch trials across the two ROIs, *F*(1,54) = 4.93, *p *=* *.03, η^2 ^= 0.084. The adjusted match trials were higher in amplitude than the adjusted mismatch trials (match: *M *=* *−0.19 μV, *SE *= 0.37; mismatch: *M *=* *−0.65 μV, *SE *= 0.33). No significant interactions were found between Trial Type and Hemisphere, *F*(1,54) = 1.31, *p *=* *.26, η^2 ^= 0.024.

#### 3.4.2 P2p (200–250 ms)

We found a marginally significant difference between adjusted match and mismatch trials across the two ROIs, *F*(1,54) = 3.03, *p *=* *.087, η^2 ^= 0.053. The adjusted match trials had higher amplitude than the adjusted mismatch trials (match: *M *=* *3.71 μV, *SE *= 0.30; mismatch: *M *=* *3.22 μV, *SE *= 0.33). No other effects were found significant, *ps *> .129.

## 4 DISCUSSION

The present study examined the integration between the approximate number system (ANS) and the symbolic number system (SNS). Specifically, the integration between nonsymbolic and symbolic formats of numbers in the human brain was examined by asking young adults to passively look at matching or mismatching numerical information in both formats simultaneously while recording their EEG. We hypothesized that ERP components that were previously found to be sensitive to numerical information should differentiate between match and mismatch trials. However, we also know from previous research that adults' estimates of nonsymbolic numerical stimuli are biased (Izard & Dehaene, 2008; Krueger, 1982; Odic et al., 2015) and that therefore an actual match between a nonsymbolic numerical stimulus and a symbolic one may not be perceived as such. To take estimation biases into account, we collected each participant's estimates of nonsymbolic stimuli similar to the ones used in the EEG task and adjusted the match and mismatch trials in the EEG task according to their behavioral estimation biases.

### 4.1 ERP symbolic integration effect

We found significant differences between match and mismatch trials over bilateral parietal scalp sites starting as early as 130 ms poststimulus when perceived rather than actual nonsymbolic quantities were taken into consideration. These findings argue for a rapid integration of numerical information across nonsymbolic and symbolic stimuli in the adult brain. The N1 and P2p ERP components have been associated with numerical processing in previous studies and have been found to be sensitive to numerical distance in both nonsymbolic and symbolic stimuli (Dehaene, 1996; Libertus et al., 2007; Temple & Posner, 1998). Our findings suggest that sensitivity to numerical information extends beyond explicit numerical processing as the participants in our study were passively viewing the numerical stimuli. Our findings also suggest that sensitivity to numerical information spans across nonsymbolic and symbolic formats of numerical information. Interestingly, the integration of nonsymbolic and symbolic numerical formats did not result in a later onset of the ERP differences than previous studies focusing on stimuli within a given format, suggesting that cross‐format numerical integration occurs as rapidly as within‐format numerical comparison. This further implies that the processing of symbolic and nonsymbolic numerical information unfolds in parallel.

However, other research suggests that the P2p ERP component may reflect the evaluation of perceptual visual features of nonsymbolic numerical stimuli. For example, Gebuis and Reynvoet (2013) showed that P2p amplitudes were modulated as a function of variation in perceptual cues of dot quantities, such as different convex hulls and densities, but that the P2p did not differentiate between different dot quantities. In the present study, we took two steps to avoid systematic perceptual confounds between mismatch and match trials. First, our stimuli were created such that each dot quantity was used both in mismatch and match trials. Second, as our adjustment shifted the alignment between the dot quantities and Arabic numerals resulting in a larger number of mismatch trials, we intentionally only used the mismatch trials based on the dot quantities used in match trials for each participant in the adjusted ERP analysis. For example, for a participant who estimated 42 dots to be 28 dots, the stimulus showing 42 dots paired with Arabic numeral 28 was considered a new, adjusted match trial. We then selected the corresponding mismatch trials as 42 dots paired with a different Arabic numeral (e.g., 42). Therefore, in both our unadjusted and adjusted match and mismatch trials, the dot quantities were exactly identical and hence any ERP differences cannot result from perceptual differences in the nonsymbolic stimuli.

A related question is whether the ERP differences we observed may stem from the perceptual differences in Arabic numerals. Indeed, in the present study the perceptual features of the Arabic numerals were not controlled, neither before nor after adjustment. However, if any perceptual difference of the Arabic numerals contributed to the P2p difference we observed after the adjustment, we should observe P2p differences in the nonadjusted analysis as well, which we did not. Therefore, it is unlikely that the integration between nonsymbolic and symbolic numerical information observed in our study results from systematic variation in perceptual features in either stimulus format. Instead, we argue for a rapid brain response that is associated with the numerical evaluation and integration of both nonsymbolic and symbolic numbers.

### 4.2 N1 and P2p amplitude differences

In previous studies, small numerical changes in an adaptation task (Hyde & Spelke, 2009) or small numerical distances in a number comparison task (Dehaene, 1996; Libertus et al., 2007; Temple & Posner, 1998) elicited higher P2p amplitudes compared to large changes or large distances, respectively. In our study, the P2p amplitude tended to be higher for match trials compared with mismatch trials. As the numerical distance between the nonsymbolic and symbolic numbers is smaller than the numerical distance in the mismatch trials, our P2p amplitude finding was in line with previous findings.

Unlike the P2p amplitude, the sensitivity to numerical information in the N1 has not been consistent across studies. One study found that the N1 amplitude was higher if the numerical distance was closer irrespective of number format (Temple & Posner, 1998), while another study found that the N1 amplitude was higher if the numerical distance was far between newly learned artificial symbols with numerical meanings (Merkley, Shimi, & Scerif, 2016). In addition, two other studies found no N1 amplitude differences between close or far conditions with neither nonsymbolic nor symbolic numbers (Hyde & Spelke, 2009; Libertus et al., 2007). Instead, the N1 amplitude seemed to be only modulated by the size of small nonsymbolic numbers (Hyde & Spelke, 2009; Libertus et al., 2007). When nonsymbolic numbers were in the small number range (<5 dots), the N1 amplitude decreases as number increases. But the N1 amplitude was not modulated by large nonsymbolic quantities. As mentioned earlier, the dot quantities in our study were exactly the same across match and mismatch trials. Thus, the N1 amplitude differences cannot be attributed to any perceptual differences between the nonsymbolic components of our stimuli. However, we were unable to also control for the perceptual features of the Arabic numerals in our stimuli. Yet, we did not see any differences between match and mismatch conditions prior to adjusting for estimation biases, which suggests that the N1 amplitude differences observed after the adjustment cannot be attributed to the perceptual differences in Arabic numerals either.

In general, the N1 component is well known for its role in visual attention (Hillyard & Anllo‐Vento, 1998; Mangun, 1995) and discrimination processes (Vogel & Luck, 2000). It is possible that the adjusted mismatch trials attracted more attention compared to adjusted match trials. Of note, the N1 amplitude did not show any differences between unadjusted match and mismatch trials, suggesting that, if any attention was involved, it was related to the perceived mismatch. This possibility needs further examination.

So far, we have shown that the N1 and P2p amplitude differences observed in our study are not likely due to perceptual features of the dot quantities or Arabic digits. However, the number of match and mismatch trials in our task was not balanced, especially after adjustment. Hence, we cannot rule out the possibility that our ERP differences may be due to the imbalance between match and mismatch trials. In fact, research that investigates general visual mismatch effects in non‐numerical visual domains revealed ERP differences in similar ERP components as the N1 and P2p in our study (Stefanics & Czigler, 2015). These ERP differences are typically evoked by a visual mismatch paradigm that consists of a stream of stimuli with different proportions of standard and deviant (oddball) stimuli. The stimuli vary from basic visual stimuli, such as shapes and gratings, to complex visual stimuli, such as faces. The general finding is that less frequent deviant stimuli are typically associated with a lower ERP amplitude than more frequent standard stimuli over bilateral occipital and occipito‐parietal sites in two components similar to the N1 and P2p around 150–250 ms poststimulus (Heslenfeld, 2003). Researchers argue that this visual mismatch effect reflects prediction errors because the deviant stimuli are presented less frequently than standard stimuli. In the visual mismatch paradigm, participants adapt to the more frequent standard stimuli and generate a prediction favoring the standard stimuli. If this prediction is violated, brain activity changes resulting in the observed ERP differences.

One study (Hsu & Szücs, 2011) examined such mismatch effect in the numerical processing domain. In this study, the authors presented two Arabic numerals simultaneously to adult participants and asked participants to judge whether the two numerals in a trial were the same or not. Two‐thirds of their total trials were mismatch trials, and one‐third was match trials. Thus, the match trials were the less frequent/deviant condition and the mismatch trials were the more frequent/standard condition. Similar to the visual mismatch effect, Hsu and Szücs found that the match (deviant) trials elicited lower ERP amplitude within the 236–328 ms time window over bilateral occipito‐parietal sites, suggesting that a general mismatch detection mechanism might have been activated rather than a number‐specific one.

In our study, we presented one‐third match trials and two‐thirds mismatch trials. The adjustment changed the number of match and mismatch trials but did not reverse the proportion of the two types of trials. Therefore, similar to Hsu and Szücs' (2011) study, after the adjustment the mismatch trials were the standard condition and the match trials were the deviant condition in our study. Thus, it is possible that participants predicted that a numerical mismatch was more likely to occur. However, unlike in visual mismatch paradigms in general and Hsu and Szücs' (2011) study in particular where the less frequent stimulus elicits the more negative ERP amplitude, we observed more positive amplitudes for adjusted match (less frequent) than adjusted mismatch (more frequent) trials during the N1 and P2p time windows. This direction of the mismatch effect suggests that our finding may not reflect a general mismatch detection process. Instead, we hypothesize that it reflects the detection of numerically mismatching information across two different number formats.

### 4.3 The role of the nonsymbolic underestimation bias for symbolic integration

Previous studies found that large quantities are likely to be underestimated (Izard & Dehaene, 2008; Krueger, 1982, 1984; Libertus et al., 2016; Odic et al., 2015). We replicate this underestimation bias in the behavioral nonsymbolic number estimation task. Importantly, we found that it was critical to take estimation biases into account to observe an integration effect in our ERP task. Before adjusting for each participant's estimation biases, we did not observe any differences between match and mismatch trials in the N1 or P2p components. However, after adjusting for individual differences in participants' estimates of nonsymbolic dot quantities, significant ERP differences emerged.

It is critical for future studies on symbolic integration to consider these underestimation biases in conjunction with other paradigms such as explicit number comparisons using different numerical formats or fMRI adaptation paradigms. In addition, it is important to further examine the origins and development of the underestimation bias for a more thorough understanding of its potential influence on the integration between the ANS and SNS across the life span.

### 4.4 Limitations and future directions

One limitation of the current study relates to the order in which the participants completed our tasks. The behavioral estimation task was always administered before the EEG passive viewing task. This order could prime the participants to integrate nonsymbolic and symbolic stimuli in the following passive viewing task. Thus, it is possible that the ERP integration effect between nonsymbolic and symbolic numbers may not have been entirely spontaneous. However, we did not provide any feedback in the estimation task and hence there was no explicit way for the participants to calibrate their estimation performance. Further studies should reverse the task order to examine whether it had an impact on participants' integration effects.

In previous studies using EEG/ERP to study numerical cognition, posterior electrodes have been found to be related to numerical processing. In our study, we selected our ROIs based on these studies (Hyde & Spelke, 2009; Libertus et al., 2007) and found the symbolic integration effect in these ROIs. Given that these posterior sites are spatially close to parietal cortex and the fact that parietal cortex, especially the IPS, has been implicated in numerical processing in other studies (Ansari et al., 2005; Fias et al., 2003; Piazza et al., 2004), it is tempting to link our findings to the function of parietal cortex. Yet, the spatial resolution of the EEG/ERP method constrains the power to make such inferences. Future studies should consider adopting methods that have both high spatial and temporal resolution. As numerical processing in the parietal cortex does not involve many subcortical structures, magnetoencephalography (MEG) could be beneficial to study the symbolic integration at the cortical level. Another potential possibility is to use an intracranial EEG method. Finally, a third way to test the involvement of parietal cortex is to incorporate lesion studies. If bilateral parietal cortices are involved in parallel but functionally different numerical processing, then patients with brain damage in unilateral parietal cortex might exhibit different patterns in their ERP waveforms in passive viewing tasks such as that used in our study. For example, patients with left hemisphere lesion might lack the symbolic integration effect in the N1 time window over left posterior sites.

Another limitation of the present study is that we concentrated our ERP analysis on the N1 and P2p time windows and our posterior ROIs. There were other time windows and electrodes where the graphed data present some evidence of the symbolic integration effect. For example, in Figure 2c,d, the waveforms for match and mismatch condition separate in the 300‐ to 400‐ms time window. In Figure 2e,f, there are electrodes located in the central regions that show the symbolic integration effect. Future analyses are needed to address the possibility of integration effects in other time windows and at other locations.

## 5 CONCLUSION

The present study tested the integration between nonsymbolic and symbolic numerical formats in the adult brain. After adjusting for participants' estimation biases inherent to the nonsymbolic format, we found greater ERP amplitudes for trials in which the symbolic and perceived nonsymbolic numerical information matched than in trials where this information did not match. This neural symbolic integration effect emerged around 130 ms poststimulus (N1 ERP component) over bilateral posterior scalp sites. Our findings suggested that the integration between the nonsymbolic and symbolic numerical information occurs rapidly but is best observed when perceived rather than veridical quantities are taken into account.
